# The Intersection of Faith and Neurodiversity: Unraveling Religiosity in Autistic Adolescents—A Critical Review Analysis

**DOI:** 10.7759/cureus.61271

**Published:** 2024-05-28

**Authors:** Aalia A Hayat, Areej H Meny, Ruth F Millman, Nabila Salahuddin, Ricky Ahuja, Waqas Sami

**Affiliations:** 1 Department of Psychiatry, Maternity and Children Hospital, Makkah Al-Mukarramah, SAU; 2 Department of Occupational Therapy, King Saud Bin Abdulaziz University for Health Sciences, College of Applied Medical Sciences, Jeddah, SAU; 3 Department of Autism and Neurodiversity, New School of Psychotherapy and Counselling, London, GBR; 4 Community Pediatric Department, Provide Community Service, Essex, GBR; 5 Autism Clinic, Independent Autism Service, London, GBR; 6 Department of Pre-Clinical Affairs, College of Nursing, QU Health, Qatar University, Doha, QAT

**Keywords:** religious experience, high functioning autism, faith, autism spectrum disorder (asd), pervasive developmental disorder, autistic teens, autistic young person, spirituality, religiosity, autistic adolescents

## Abstract

This literature review aims to explore religiosity, faith, and related beliefs in autistic adolescents. The term religiosity was used interchangeably with various related concepts such as faith, spirituality, and religious beliefs, and a broader, multifaceted approach encompassing the cognitive, subjective, social, cultural, and emotional domains of religiosity is analyzed in this population subgroup. In alignment with the neurodiversity paradigm, this review endeavors to adopt an inclusive lens toward autism spectrum conditions, appreciating the spectrum of cognitive and behavioral differences and highlighting the importance of recognizing strengths and challenges alike, reflecting the nuanced discourse surrounding neurodiversity and autism spectrum conditions. However, terms such as "high-functioning autism" and "disorder" were used where needed to reflect the journals included in the review. A systematic search was conducted by accessing academic search engines such as APA PsycInfo, APA PsycArticles, APA PsycTests, and PubMed. Only peer-reviewed articles written in English and performed on human subjects were included using strict inclusion and exclusion criteria.

Several recurring themes were identified from the 13 articles selected after review for relevance and quality. The most important finding was the association of different terminologies and features while exploring “religiosity in autism.” Thirty-nine key themes were identified, which were grouped into six major themes. These were religious faith, spirituality, and its expression in autistic adolescents; religious behaviors and practices of autistic adolescents; cognition and religion in autistic teens; social and cultural influences on religiosity in autistic young ones; parents' and carers' influence, perspectives, and experiences about faith and spirituality on autistic adolescents; and perceived benefits of faith to autistic teens: parents and adolescent perspectives.

Looking at the concept of religiosity and spirituality as a whole, it can be inferred from the available research included in this review that religiosity (cognitive abilities, behaviors, and experiences) in a subset of autistic adolescents (high-functioning autism) might not be significantly subdued as compared to neurotypical adolescents. However, there is not enough research to conclude the same or the opposite for autistic adolescents in general. When found, reserved religiosity could be attributed to a plethora of factors, and decreased mental ability or mentalization, empathy, or imagination did not seem to be the sole or primary predictors or contributors to religiosity. The role of culture, parents, carers, and religious affiliations was significant and might be a stronger contributor to religiosity and its expression than other previously argued predictors like mentalization.

Many autistic teens and their carers regard religiosity and spirituality as essential domains in their and their children's lives, want their children to be given opportunities to be a part of religious groups and affiliations, and look forward to government, religious, and healthcare authorities actively supporting them in this domain. The findings call for policymakers, religious leaders, and stakeholders to devise strategies for inclusion and support for autistic adolescents. The possible role of religion as a resource and coping strategy for these children and their families is worth exploring.

## Introduction and background

Adolescence is a transitional period characterized by change. Along with hormonal, physical, and emotional development, there is a shift from childhood to adulthood. Many adolescents find this difficult, often becoming alienated when entering the adult world [1]. Developmental science does not typically investigate religion and spirituality [2]. Researchers have maintained that spirituality is an integral part of everyday human lives and is relatively central to adolescent development [2-4]. Recently, there has been an increasing interest in the interaction between religiousness, spirituality, and disease [5]. Neurodevelopmental disorders are recognized as one of the leading causes of morbidity in children and contribute significantly to the global burden of disease (GBD) [6].

According to a recent review, approximately one out of every 100 children has autism spectrum disorder (ASD), and there has been a continuous increase in the prevalence estimates over time [7]. The etiology is multifactorial; however, brain imaging studies do report neurobiological abnormalities in autism, like diminished integrity of gray matter neurons and lowered glutamatergic activity in the cortex and cerebellum [8]. It is also observed that religious and spiritual attitudes are affected in patients with various neurobiological abnormalities since the integrity of neuronal pathways is integral to forming and understanding various concepts, including those around spirituality and religiosity. However, the exact mechanism is unknown [5].

Definitions and terminologies

Determining what religion is can be complex [9]. The terms religiosity and spirituality will be used interchangeably in this research paper. Researchers have used various terminologies to define the neurodivergent conditions "autism" and "autism spectrum disorder." The review supports a neuroaffirmative approach, promoting a more inclusive and accepting view. However, various other terminologies will be referred to when needed while referring to the work of various researchers included in the review.

Attempting to describe the broadest range of religiosity, Bergan and McConatha defined religiosity as having multiple dimensions related to various religious beliefs and practices [10], and for research purposes, we will keep referring to this definition.

“Religiosity refers to the various dimensions associated with religious beliefs and involvement. More recent studies have stressed the importance of evaluating religiosity as a multidimensional concept focusing on subjective, cognitive, behavioral, social, and cultural components.”

Religiosity and autism

Autism [11] and religiosity [12] are both complex and multifaceted phenomena. Various studies have explored the relationship between autism and religiosity, some suggesting that autistic traits might be negatively associated with religiosity [13]. In contrast, others cite negligible differences in the religious behaviors or cognitions of these individuals when compared to neurotypical (NT) individuals [14].

The GBD study revealed that over one million children living in South Asia and Sub-Saharan Africa are autistic. As of 2018, India has the highest number of autistic children (851,000), followed by China (422,000), Nigeria (207,000), Pakistan (172,000), and Indonesia (159,000) [15]. Most of these areas and regions favor or prefer religion in one form or another. For example, Pakistan and Nigeria are declared as Islamic states, and Indonesia, though it declares itself a secular state, harbors the world’s largest Muslim population [16].

Although currently there are very few autism prevalence studies from the Middle East and Africa, they reveal that the prevalence of autism ranges somewhat between 20.35/10,000 in Oman [17] to 114/10,000 in Qatar [18] and 290/10,000 in East Nigeria [19], which is comparable to or higher than the global prevalence of 230/10,000 [20] despite arguments that autism is underrepresented in these regions due to issues of inadequate diagnostic services [21], poor adaptation of diagnostic criteria with regard to cultural differences in behavior, and undersampling due to lack of reliable national data [22].

The study of religion shows that religious leaders and religion play an important role in the lives of many people, including those studied in the UK [23,24]. Although sometimes religious beliefs can affect whether families are seeking a diagnosis or not, they were seen as providing emotional and social support to the cases and families of autistic people in many instances. Reportedly, religion gave the family the strength to carry on and accept the diagnosis in some instances [23]. Similarly, a study on people of color, Asians, and ethnic minority groups in the UK revealed that belief systems played a significant role in autism diagnosis acceptance [24]. It was postulated that faith might give the parents of autistic children strength and hope for the future [23].

Despite the observed supportive impact, the misconceptions about autism held by different religious faiths are common and sometimes unhelpful. They could have a negative outcome, affecting the well-being of autistic people and their families [24]. In extreme cases, it has been suggested that autism is caused by demonic spirits or magical beings, which might alarmingly give rise to harmful treatment practices like exorcism [25]. It is not unheard of that some religious figures stress that parents should rely solely on prayer to treat the disorder [24]. Some places of worship might limit the inclusion of autistic people, giving them a feeling of isolation [26].

A literature search reveals that there is little research on the religious beliefs of autistic individuals [27]. What little there is suggests that autistic people vary in their beliefs, with some being more firmly attached to religious faith than others [28]. One study found that people who have more features of autism are less likely to believe in God than those who do not have such traits [29].

A study by Stuger [30] has shown that autistic individuals can hold strong religious beliefs and are often more faithful than their nonautistic peers. Additionally, it postulated that autistic individuals may tend to stick to traditional religious beliefs than their nonautistic counterparts [30]. Yet another study found that autistic people may have a different relationship with religious beliefs than nonautistic individuals [31]. Moreover, some studies have suggested that creativity can be seen as a field of relationship between God the Creator and autistic people [32,33], with each person bringing unique perspectives to the discussion. Although researchers have found that religion might offer a framework for comprehending existential anxieties and daily life's ups and downs in NT individuals [34], no concrete evidence suggests the same for autistic individuals.

Researchers focusing on the religious perspective of high-functioning autistic (HFA) individuals proposed that, instead of there being a deficit, HFAs' thinking styles are on a continuum with NTs [35]. Some researchers concluded that atheism and agnosticism are supported more frequently by individuals with HFA than by NT individuals. The authors likened them to scientists with a high rejection rate for religious beliefs [36]. Some believed that the HFAs appreciated socially welcoming religious communities, while others considered them rigid and doctrinaire [37]. Despite the variety of opinions in this regard, most of the work implies that research into the beliefs of autistic individuals is essential in understanding the potential impact of religious beliefs on their lives [36].

Researchers have maintained that spirituality is an integral part of everyday human life [2] and is relatively central to NT adolescent development [4]. During this time, spiritual assessment, examination, and consideration are often undertaken [38]. According to research, religious discussions happen during late adolescence in NT individuals. Additionally, early adolescence is the period when religious commitments are formed [39]. Furthermore, religion is considered an essential component of identity [40], with religious identity being formed in late adolescence [41-48]. Considering that adolescence is a distinct period of transition with its unique challenges and in NT adolescents, a period when religiosity [49-57] and religious identity are being formed [58-62], understanding the presence of religion and related concepts and their significance, if any, in this particular age group is of paramount importance. Not much published data throw light on the degree of religiosity that this particular patient population holds.

## Review

Research methodology

The final search was done using the terms:

1. Autism: autism OR Asperger OR "pervasive developmental" OR Kanner* OR "high functioning autism" and

2. Religion: religion OR spiritual OR faith OR secular OR atheist OR Religious experience OR "religious belief" OR "belief system" OR "religious cognition" OR prayer

3. Adolescent: adolescents OR teenagers OR young adults OR teens OR youth OR young people

Inclusion and Exclusion Criteria

The research articles whose central theme was spirituality with the debarment of religious dimensions were not included because a person can be spiritual without being religious. For this research, the focus was on religiosity, and it included the papers with spirituality as a theme in dimensions of religiosity while excluding the ones studying spirituality individually [42]. The search was carried out from 1995 to date. The preliminary exploratory literature review and research suggested that since the middle of the 1990s, the amount of research on religiosity/spirituality and health has increased [43], so the search was limited to this time frame. Only peer-reviewed articles written in English and performed on human subjects were included.

Information Sources and Search Strategy

**Figure 1:**
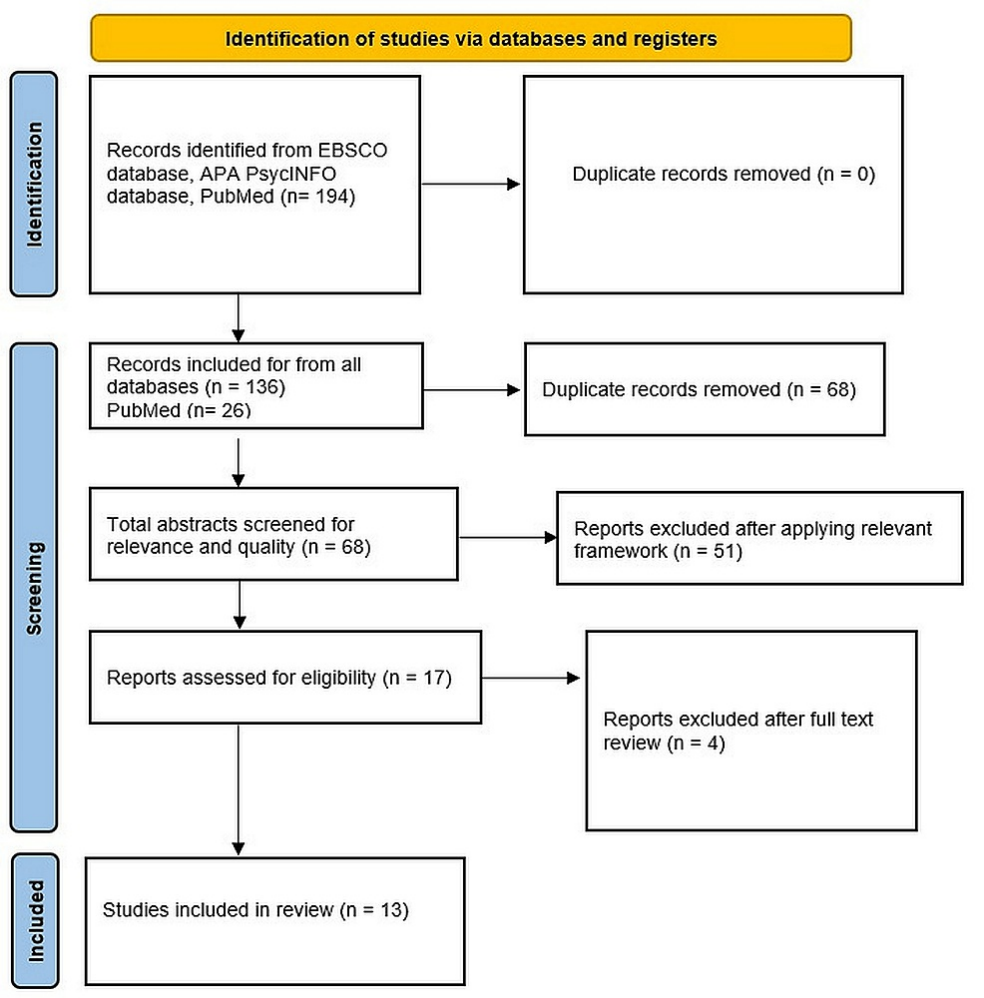
PRISMA flow diagram PRISMA: Preferred Reporting Items for Systematic reviews and Meta-Analyses; EBSCO: Elton B. Stephens Company

The academic search engines accessed were APA PsycInfo, APA PsycArticles, APA PsycTests, and PubMed. An additional Google Scholar search was conducted to include any missed articles, details of which are provided in the search strategy section and summarized in Figure 1.

Screening of Records

The initial search on APA PsycInfo, APA PsycArticles, and APA PsycTests returned 117 records (after removing duplicates), while that on PubMed returned 77. The articles after applying limiters were (n = 58). The number of articles included for basic screening was n = 136, and for duplicate record removal, it was n = 68. None was returned from APA PsycTests. Table 1 displays search results and search strings.

**Table 1:** APA PsycInfo, APA PsycArticles, and APA PsycTests search results APA PsycInfo: 35; APA PsycArticles: 2; no duplicates

	Search strings
S1	autis* OR Asperger* OR "pervasive developmental" OR Kanner* OR "high functioning autism"
S2	religio* OR spiritual* OR faith OR secular* OR atheis* OR Religious experience OR "religious belief" OR "belief system" OR "religious cognition" OR prayer
S3	adolescen* OR teenager* OR young adults OR teen* OR youth OR young people
S4	S1 AND S2 AND S3
S	(autis* OR Asperger* OR "pervasive developmental" OR Kanner* OR "high functioning autism") AND (religio* OR spiritual* OR faith OR secular* OR atheis* OR Religious experience OR "religious belief" OR "belief system" OR "religious cognition" OR prayer) AND (adolescen* OR teenager* OR young adults OR teen* OR youth OR young people) Limiters - Peer Reviewed; Publication Year: 1995-2023; English; Age Groups: Adolescence (13-17 yrs); Population Group: Human; Document Type: Journal Article

This yielded 68 articles for further screening, as shown in Table 2.

**Table 2:** PubMed search results

	Search strings
S1	autis* OR Asperger* OR "pervasive developmental" OR Kanner* OR "high functioning autism"
S2	religio* OR spiritual* OR faith OR secular* OR atheis* OR Religious experience OR "religious belief" OR "belief system" OR "religious cognition" OR prayer
S3	adolescen* OR teenager* OR young adults OR teen* OR youth OR young people
S4	S1 AND S2 AND S3
S	(autis* OR Asperger* OR "pervasive developmental" OR Kanner* OR "high functioning autism") AND (religio* OR spiritual* OR faith OR secular* OR atheis* OR Religious experience OR "religious belief" OR "belief system" OR "religious cognition" OR prayer) AND (Adolescen* OR teenager* OR young adults OR teen* OR youth OR young people) Filters applied: Case Reports, Classical Article, Clinical Study, Clinical Trial, Comparative Study, Controlled Clinical Trial, English Abstract, Evaluation Study, Historical Article, Interview, Meta-Analysis, Multicenter Study, Observational Study, Personal Narrative, Pragmatic Clinical Trial, Randomized Controlled Trial, Review, Systematic Review, Twin Study, Validation Study, Humans, English.

All returned records, along with complete citation details and an abstract, were transferred from the database to Zotero, a program for managing bibliographies, where duplicate records were eliminated. After removing duplicates, the total number of articles to be screened was 68.

Screening of Article Abstracts

To decide whether to include relevant articles, a relevance decision scheme was formulated to screen the 68 article abstracts from the initial search. Each abstract was screened and either excluded or included accordingly (Table 3).

**Table 3:** Abstract screening algorithm

S. no.	Screening step	Decision
1.	Is the study a research article authored in English and released in 1995 or later?	If no, exclude; if yes, move on to the next step.
2.	Is the central theme of the study religiousness, religiousness/secularism/atheism in autism?	If no, exclude; if yes, move on to the next step.
3.	Does the study focus on spirituality individually and not as a part of broader religiosity?	If yes, exclude; if no, include.
4.	Does the publication focus on young people and adolescents?	If no, exclude If yes, or a mixed population group, including adolescents, move on to the next step.

About 51 articles were excluded because they were not in English, their central themes were not relevant, two were book chapters, and others did not target the specified population group. Seventeen final articles were selected for a full-text review to assess their quality and determine inclusion.

Full-Text Review for Relevance and Quality

The full text of the remaining 17 records was located and reviewed. It was reviewed for relevance and quality using questions detailed in Table 4.

**Table 4:** Algorithm for screening full texts for relevance and quality

S. no.	Screening step	Decision
1.	Is the article peer-reviewed?	If no, exclude; if yes, move on to the next step.
2.	Does the research sufficiently describe religiosity and related concepts in autistic individuals?	If no, exclude; if yes, move on to the next step.
3.	Does the research have a specified methodology section describing the data collection and analysis procedure?	If no, exclude; if yes, move on to the next step.
4.	Is evidence provided to back up the results and conclusions?	If no, exclude; if yes, include it in the final analysis.

After reading the full texts, four further studies were excluded. One addressed spirituality exclusively, one targeted disabilities broadly, and two did not address the relevant theme. This yielded a total of 13 studies for final inclusion in the analysis. Figure 1 provides a Preferred Reporting Items for Systematic reviews and Meta-Analyses flow diagram of the search and screening processes.

Assessment of Study Quality

Studies yielded a mix of various methodologies, ranging from surveys and comparison analysis to qualitative interviews and mixed-method studies. All articles included were peer-reviewed. The articles included a good mix of perspectives from the autistic adolescents and the parents’ and caregivers' reports (Table 5).

**Table 5:** Characteristics of articles included in the review

S. no.	Journal article	Study	Article category	Participants	Peer-reviewed
1.	Rethinking autism, theism, and atheism: bodiless agents and imaginary realities	Visuri, 2018 [31]	Mixed methods study	Adolescents	Yes
2.	Religious cognition among subjects with autism spectrum disorder (ASD): defective or different?	Ekblad and Oviedo, 2017 [32]	Cross-sectional survey	Adolescents	Yes
3.	Religious cognition and behaviour in autism: the role of mentalizing	Reddish et al., 2016 [14]	Cross-sectional comparison study	Adolescents	Yes
4.	Religious and spiritual expressions of young people with intellectual and developmental disabilities	Carter and Boehm, 2019 [49]	Cross-sectional descriptive study	Parents	Yes
5.	Religion and positive youth development: challenges for children and youth with autism spectrum disorder	Sullivan and Aramini, 2019 [28]	Qualitative interviews	Parents	Yes
6.	Mentalizing skills do not differentiate believers from non-believers, but credibility-enhancing displays do	Maij et al., 2017 [45]	Cross-sectional survey	Adolescents	Yes
7.	Parents’ perspectives on the participation of their children with autism in Sunday school	Howell and Pierson, 2010 [50]	Cross-sectional semi-structured telephone interviews	Parents	Yes
8.	Sensory supernatural experiences in autism	Visuri, 2020 [48]	Mixed methods study	Adolescents	Yes
9.	Quality of life for transition-age youth with autism or intellectual disability	Biggs and Carter, 2016 [46]	Cross-sectional survey	Parents	Yes
10.	In their own words: the place of faith in the lives of young people with autism and intellectual disability	Liu et al., 2014 [44]	Qualitative interviews	Adolescents	Yes
11.	Mentalizing deficits constrain belief in a personal god	Norenzayan et al., 2012 [29]	Cross-sectional observational study	Adolescents	Yes
12.	Children with autism: quality of life and parental concerns	Lee et al., 2008 [47]	Cross-sectional study	Parents	Yes
13.	Youth ministry, religious education, and adolescents with disabilities: insights from parents and guardians	Jacober, 2010 [51]	Qualitative interviews	Parents	Yes

Results and findings

Several key findings emerged from this research, the most important of which was the association of different terminologies and features with "religiosity in autism." It was represented under many titles such as "Autism, Theism, and Atheism" [31], "The Place of Faith in Autism" [44], "Mentalizing Deficits, Cognition, and Autism" [14,45], "Quality of Life in Autism" [46,47], "Sensory supernatural experiences in autism" [48], "Religion and Positive Youth Development" [28], "Religious and Spiritual Expressions of Young People with Disabilities" [49], "Children with Autism in Sunday School" [50], "Religious cognition in autism" [32], and "Youth Ministry and disabilities" [51]. Regardless of the variation in terminology, the studies presented findings that highlight the importance of research on religiosity in autistic adolescents.

Measures of Religiosity

The researchers used a wide variety of tools to capture these themes depending on their research questions and study feasibility. Some measures were parent reports, surveys, or interviews (6/13), while the rest collected data directly from autistic adolescents. Six studies used open-ended questions to lead qualitative interviews tailored according to the participants and study theme. One researcher used an innovative method by asking the participants to prepare their interview by taking photographs of whatever objects, places, persons, etc. they considered were related to religion or spirituality in their lives and then bringing them to the interview and talking about them [31]. The remaining researchers used various measures, such as modifying and extracting related items of religiosity from preexisting validated tools while assuring their internal validity [14,49], using a self-constructed nonvalidated religiosity measure [45], using a validated short form [46] of the "Santa Clara Strength of Religious Faith Questionnaire" [52], and extracting relevant data from a national survey of children’s health [47] or an adapted version of the "intuitive religious belief scale" [53].

Key Themes

After analyzing the articles in detail, the main findings were extracted, and descriptive data were organized in table form. The key results were extracted. Thirty-nine key themes were identified and grouped into six major groups for discussion purposes. Most of the themes were recurring and overlapping and could be placed in multiple groups. The following are the six main groups, each comprising various subthemes.

1. Religious faith, spirituality, and its expression in autistic adolescents.

2. Religious behaviors and practices of autistic adolescents.

3. Cognition and religion in autistic adolescents.

4. Social and cultural influences on religiosity in autistic adolescents.

5. Parents' and caregivers' influence, perspective, and experiences about faith and spirituality in autistic adolescents.

6. Perceived benefits of faith to autistic adolescents: parents and adolescent perspectives.

Discussion

The review highlighted an interesting array of findings regarding religiosity in autistic adolescents. The most prominent finding was the vastness of religiosity, related concepts, terminologies, and the interplay of various domains of life and the sciences, in the lifespan of autistic adolescents. Observable external religious behaviors and practices, as well as deep-rooted internal feelings and personal experiences, were studied in the target population. The findings demonstrated that numerous factors interplayed to shape the experience and manifestation of religiosity in this population group (Table 6).

**Table 6:** The narrative matrix for the synthesis of quantitative studies CRED: cognitive reflection and epistemic development; GFT: geometric figures task; AQ: autism quotient; IQ: intelligence quotient; TD: typically developing; HFA: high-functioning autism; ID: intellectual disability; NSCH: national survey of children's health; ADHD: attention deficit hyperactivity disorder; ASD: autism spectrum disorder; NT: neurotypical; EToM: existential theory of mind; AS: autism spectrum; IRI: interpersonal reactivity index; SPRED: specialized program for children with autism

Reference	Study design	Parameters/tools used to study	Sample size and population	Results	Strengths	Limitations
Carter and Boehm, 2019 [49]	Observational, cross-sectional, and parent-reported measures	Involvement in congregational activities, engagement in spiritual practices, and strengths of religious faith	Parent age: 31-72 years; adolescents age: 13-21 years; mean: 16.6; N = 440; 55.2% = autism	Specific for autism: fewer congregational activities were associated with the presence of an autism label and lower ratings for parents' strength of religious faith. Lower ratings for youth’s strength of religious faith were associated with the presence of an autism label and lower ratings for parents’ strength of religious faith. No association between autism label and spiritual practices was found	Observable activities and practices, targeting various factors especially parents' perception about adolescents' faith and also associations with various child and parent factors, and a large sample size	Mixed population idd+ autism, parent-reported, mixed age range: adolescents and young adults, and mean age was 16.4 years in Tennessee, with higher religiosity (82%)
Maij et al., 2017 [45]	A mix of five individual studies; study 5 fits the inclusion. Observational and cross-sectional comparison	Autism quotient, religiosity, religious behaviors, and CRED (cultural learning variables) were measured through questionnaires and scales. GFT using videos followed by rating those videos as instructed) was done to measure intentionality (the measure of mentalizing)	33 adolescents with autism. 29 controls 13-18 years	The AQ was higher in the autism group. Groups did not differ on religiosity, religious behaviors, and CRED. No difference was observed for intentional videos about GFT. They attributed less intentionality to random and mechanical videos	The association between mentalization and religiosity was studied using various measures and techniques. The study is self-reported	28 (autism) vs. 17 males (control), not longitudinal, and religiosity questionnaire was not validated
Reddish et al., 2016 [14]	Observational and cross-sectional comparison	Mentalizing measures (false belief and mentalizing tasks, strange stories), autism quotient, IQ tasks, and self-report questionnaires on religious cognition and behavior	N = 19; matched comparison group: 19; age: 12-30/31 years; relationship measures were controlled for age	No statistically significant difference in religiosity between the two groups was found. Lower mean scores on mentalizing were not a significant predictor of any of the religiosity measures. The only significant difference was that TD found praying in general more attractive than HFA. Greater attraction to prayer was associated, however, with lower deficits in social skills and could be linked to difficulties in social interaction in general. A marginal significance of negative correlation with nonanthropomorphic god concepts (lower mentalization and greater tendency to agree with abstract qualities of god) was found	Investigated the role of mentalization in religion by comparing HFA and TD individuals, self-reported, and tedious method to match the controls. Used multiple mentalizing measures. Seven religiosity domains were assessed using different questions adapted for the study, and internal validity was assessed for the individual domains	Mixed population age (however, associations were calculated after controlling for age). Only one type of ritualized behavior-scripted prayer was measured. All the participants except one identified as religious in the HFA group
Biggs and Carter, 2016 [46]	Observational, cross-sectional, and parent-reported comparison	Quality of life (QoL) domains, religiosity measures, 13 predictor variables like age, gender, ethnicity, challenging behaviors and gender using parent-reported questionnaires	Parent/guardian mean age: autism group: 47.1; youth age: 13-21; total N = 3,890; autism: 232; adolescents with autism (109) followed by 71 from 16-18 years and 52 from 19-21 years	Strength of faith and autism had a statistically significant negative correlation. Faith had a strong positive association with all five domains of QoL except the physical one. The strength of faith in the sample with autism was 2.4/4. (A comparison with the normative adolescent sample was not provided.) In the original scale development, the mean was 2.71 (Plante et al., 2002 [52]—sample 1). Higher ratings on faith predicted increased scores in the “social support and peer” and "autonomy and parent relations” domains. Youth with autism had statistically significantly lower values in the domains of “ physical,” psychological,” and “social support and peers” of QoL when compared to the normative adolescents' sample	The correlation between strength of faith, autism, and domains of quality of life was investigated. Validated measures for religiosity and QoL were used. A comparison was done with the normative adolescent national sample	A mixed sample of autism and ID (data were analyzed for individual subsets, though). A mixed age range was used without controlling for age during data analysis. However, there was no correlation between age and faith in the bivariate correlation analysis
Lee et al., 2008 [47]	Cross-sectional, observational, and parent-reported data from a NSCH comparison with healthy controls and ADHD group	QoL questions, including religious service attendance and parental concerns	N = 173 for 12-17 years; controls: 23,768. Data were analyzed separately according to age ranges	Parent-reported carer burden was significantly higher in the autism group compared to both groups. Compared to the control group, the autism group attended religious services about 50% less frequently. Compared with unaffected control children, parental concerns in all five domains (achievements, self-esteem, stress coping, learning difficulty, and being bullied) were significantly higher in children with autism. There was a statistically significant difference between the unaffected group and the autism group in the restricted, stereotyped behaviors (religious service attendance) in one per year to less than one per week (22% vs. 25.4%) and one per week or more (41.6% vs. 56.0%)	Data from all 50 states and the District of Columbia were obtained by random digit dialing and large sample size	One question assessment with three options was extracted for religious attendance, control was matched for age and absence of disability only, the diagnosis was parent-reported, IQ was not done, cross-sectional design, and information bias (parent-reported)
Jacober, 2010 [51]	Cross-sectional, observational, and exploratory qualitative interviews	Semistructured interviews with families of adolescents having a range of developmental disabilities, including autism	Seventeen Christian families with adolescents (grades 7–12)	The main themes identified were 1) teaching adolescents about Christ, 2) community and connection, 3) positive and negative encounters with ministry, 4) what teenagers offer to a community, and 6) words of wisdom from families. The parents of adolescents with disabilities in this study wanted their children to know God and experience the fellowship of other believers to the best of their child’s ability. “Peer buddy system” and “Capernaum” were repeatedly quoted as positive experiences, but overall negative experiences surpassed the positive experiences. Families expressed views that they were not allowed in the service, and some said that perhaps there was no benefit to them being there. Others expressed that their children liked getting involved in prayers and choir, and were empathic in church	Focus on the first-hand experience of religious education, both implicit and explicit, for adolescents with disabilities and its impact on families. There is a clear desire on the part of the families of youth with disabilities for inclusive religious education. There is also a clear awareness of the barriers to just such a hope. Many parents and guardians recognized the blessing their child was and could be. They spoke of the depth of relationships their child was able to maintain. This element of relationship spoke to the incarnation and the presence of Christ in the lives of those in a Christian community. It spoke to that which was beyond religious education, reflecting instead internalization	One religious group, mixed data from all disabilities, and the account of families
Howell and Pierson, 2010 [50]	Cross-sectional, observational, ll, parent semistructured interview	Semistructured, telephonic interview with 25 open-ended questions	Four families of children with autism were interviewed. Data were shared separately from each interview. Data from one family with adolescents were included	The children from this family were not included in Sunday school. Instead, they went to an alternate “Sunday school in modified settings,” which was held in a different location, with peers without disabilities. The modified lesson was prepared by the mother of these children and the children engaged in preferred activities (puppets, bubbles, and crafts) during the lesson, which was held in a playground. Seven typical peers and three children and adolescents with autism interacted during this lesson. The children did not play or interact with the same peers outside the church. The mother reported that her children with autism loved attending church as the modifications made were geared toward their interests and strengths	The Sunday church inclusion and church experience of children with autism were addressed. Barriers and challenges to inclusion were discussed	Small sample size, parent-reported, and cross-sectional
Norenzayan et al., 2012 [29]	Observational, cross-sectional, and comparative study	IQ-Kauffman brief intelligence test. Belief in God using “Intuitive Belief in God Scale”—self-reported (modified), parent versions of AQ, and mentalizing (empathy quotient)—short version	ASD: N = 12; mean age: 13.7 years. NT (neurotypical): N = 13; mean age: 12.6 years. One female in each group. All the parents identified as religious except for one in the autistic group	Autistic participants were only 11% as likely as neurotypical controls to strongly endorse God (p = 0.046), and IQ was unrelated to belief (p = 0.64). In this logistic regression model, only mentalizing was a significant predictor of belief in God: for each standard deviation decrease in mentalizing, participants were only 21% as likely to strongly endorse God (p = 0.01), and IQ was not a significant predictor (p = 0.33)	Study 1 compared religious belief in a sample of adolescents with autism with a neurotypical sample matched on relevant sociodemographic characteristics. The study looked at possible predictors of religiosity such as mentalizing and IQ	Small sample size and parental reported mentalizing measures (AQ and empathy quotient)
Liu et al., 2014 [44]	Cross-sectional, observational, and qualitative interviews	In-person, semistructured interviews, with 15 questions and 12 follow-up probes. Seven questions about personal lives, four questions about involvement in faith community and spiritual expression, four questions about self-perception in the context of faith	20 participants with ID and ASD. Twelve had ASD, of which eight were between 13-17 years. The remaining three autistic youths were 19 years old. Collective as well as individual data were provided for each participant	Themes were identified, and collective expression, as well as individual expression and verbatims, were provided. Participants in the study spoke vividly about the importance of faith in their lives. Almost all of the youth said they engaged in the practice of prayer; half addressed how they expressed their faith through particular beliefs and behaviors; and three-quarters shared some of the ways their faith had a positive impact on their lives. Almost all but one of the young people spoke about prayer as a central spiritual activity and an important aspect of their faith. All participants said that their families were committed to their congregation and would bring them there. The most frequently addressed theme was how their faith was beneficial to them. Several youths considered their disability as their strength or a gift to be used. Only two participants, however, saw it as a condition to be healed or relieved. All eight adolescents with ASD attended worship services weekly except one, who did so monthly. Five out of eight attended Sunday school weekly or monthly. Three were usher greeters or altar servers, from monthly to yearly. One sang in choir monthly. Three attended prayer meetings yearly and seven attended the sacraments weekly to monthly. Half said that they expressed their spiritual affiliations through social gatherings, while half described local outreach as their medium of spiritual expression. All eight participants committed to having different religious affiliations	Expressions of the place of faith in the lives of youth with intellectual and developmental disorders in their own words were provided. Their perspective on how they viewed their disability in the context of their faith was explored. Participants from varied religious affiliations were included. Verbal accounts of participants were included. Some descriptive data and frequencies about spiritual expressions were also tabulated separately for each participant	One state—Tennessee, all parents were identified with religious groups, and all participants were identified with religious groups. Participants did not include adolescents without speech as their main form of communication. IQ was not known. Longitudinal design. The level of disability was not mentioned
Ekblad and Oviedo, 2017 [32]	Observational and cross-sectional comparison	Two studies: A. ASPICE quiz, modified with 40 additional questions related to religiosity from another validated tool. Data were collected through an online survey. B. Ad hoc questionnaire assessing EToM to ask about levels of religious practice	A. The age range was not mentioned, and the average age was 31 years with an SD of 14, suggesting the presence of adolescents. 62% NT, 32% self-diagnosed ASD, and 6% formal dx ASD. B. Four samples were taken from schools in Spain, with one (1b) having a mean age of 15.3 years, while the remaining had children between means of 10.9-11.6. Each sample had one boy professionally diagnosed with autism in class	A. Statistically significant findings were that the autistic sample scored higher on the factors of spirituality, religious practice, and negativity than both the self-diagnosed ASD sample and the neurotypically developing and lower on “ rationality” than both comparison groups. B. The perception of God as a protective agent was quite high in all the samples and either declined with age or became negatively correlated with age (the students 15 years of age perceive a less positive sense of providence). AS participants scored average or above average in factors of EToM, such as God knows us, helps and protects, God guides our conscience, God neglects, is absent, religious practice, and sense of providence despite the odds	B. EToM works more vividly until a certain age, around 12-13 years, when individuals rather naturally attribute some abilities to influence life events, especially in a positive way, to incorporeal and transcendent agency and that the ability or tendency to attribute agency to supernatural beings decreases as individuals grow	B. Convenience sampling, unvalidated tool, small size, and strong religious background/Christian schools
Visuri, 2018 [31]	Observational, cross-sectional, and qualitative interviews with a qualitative arm: comparative matched control group for comparing empathic concern only	First phase: self-report surveys, psychometric tests, IRI (empathy), and a religiosity questionnaire. Those who affirmed an active element related to religiosity/spirituality were included. In the second phase, a photographic life-story interview was designed for the study, assigning the participants the role of the expert, preparing their interviews by taking photos of aspects deemed relevant regarding spiritual and religious experiences, and then guiding the researcher through the photos. In the final phase, qualitative in-depth interviews were conducted where the participants presented their photos, and the researcher inserted questions according to a semistructured interview model, where needed, to clarify the narrative	16-21 years old, formally diagnosed, HFA from a Swedish school; N = 17 and N =16 for the quantitative arm; 12 males and five females	Three main themes identified were mentalization, imagination, and internalization of popular culture. Subthemes identified were communications with bodiless agents, meaning-making from events, shifting between multiple realities (like daydreaming, games, and books), relations (like imagining to be part of adventures), and coping using these phenomena (like solving problems in fantasies). Quantitative results, by comparing scores on IRI with those of a comparison group, showed no statistically significant difference in empathic concern scale scores (the autism group scored slightly higher). The result was in line with the qualitative interview. Many participants reported that communicating face-to-face was both difficult and exhausting. All participants gave examples of mental states in invisible agents, such as gods, angels, and spirits (and other imaginary characters), and their reasoning suggested that complexities may be bypassed when considering intentionality in these agents. All 17 described interactions with one or more bodiless agents (invisible companions). Eleven out of seventeen described "God” as an invisible companion. Several of the participants who described themselves as spiritual were referring to alternative, superhuman characters. Twelve out of seventeen participants could be compared to the “fantasy-prone type,” where most were described as being a part of various imaginary worlds that often were inspired by popular culture and were able to transition between “imaginary” and “reality” voluntarily	First-person perspective on autism, religion, and cognition is explored. A variety of religious orientations were represented. The role of mentalization and imagination in autistic individuals' reasoning about invisible agents was targeted. Multiple modalities and unique methodologies were incorporated to explore these complicated themes. The author postulated that imaginative autistic individuals may be attracted to religious and imaginative contexts and the agents involved in these because of qualities that facilitate social interaction (like more aligned social and emotional cognition, fewer cues to interpret, and a self-regulated pace). Participants described the bodiless agents as predictable, emotionally coherent, and benevolent, contrary to the popular notion that autistic individuals might find mental representations of superhuman agents perplexing	A mixed sample of youth was analyzed, and themes were derived from a collective sample
Visuri, 2020 [48]	Observational, cross-sectional, and mixed methods study	Psychometric tests, supernatural experience subscale, and autism quotient were done for both groups, autism and comparison. Photographic life-story in-depth qualitative interviews were done with autistic individuals only to examine the attribution of supernatural agency	16-21 years old, formally diagnosed, HFA from a Swedish school; N = 17; 12 males and five females. Comparison group matched according to age, gender, and philosophy of life	The mean score for religious experience was significantly higher for the autistic group than the matched nonautistic comparison group. It was also more varied. Examining the distribution between the different items, the comparison group has a sample maximum of four types of experiences, while nine of the autistic individuals report 4–13 types. The Autism group reported a broader variety of experiences, with most of the felt presence of a spirit (f = 13) and “other” supernatural experiences (f = 9). Autistic individuals scored higher on the felt presence of God compared to the nonautistic group. None of the comparisons described experiences in an out-of-body experience, other supernatural experiences, or telepathic contact. The comparison group scored slightly higher in one domain only: things appear predestined. Qualitative analysis revealed two main themes of somatosensory experience (nine sensed the presence of invisible bodies and 18 had visual, tactile, auditory, and olfactory experiences) and mental constructs such as dreams and divine agency. This study illustrated how cultural and generational specifics come into play in the attribution of supernatural agency. The participants preferably ascribed agency to invisible agents that are found outside organized religions	Unusual somatosensory experiences are frequent among (a subset of) individuals on the autism spectrum and were more than a matched nonautistic sample. Fourteen out of seventeen participants responded that they have had at least one somatosensory experience that is labeled in supernatural terms. Participants in the current sample appear to use supernatural attributions as a coping mechanism. The study hypothesized how Western secularization and occulture might have affected the process of supernatural attributions. The participants preferably ascribed agency to invisible agents that are found outside organized religions, a choice which may be explained by the media in which young Swedish participants encountered supernatural Ideas	Small sample and mixed data. Did everyone have a religious affiliation?
Sullivan and Aramini, 2019 [28]	Cross-sectional, observational, and qualitative interviews	Semi-structured, face-to-face interviews with parents and caregivers of children with autism. Questions fell into three major categories: background information and initial experience with autism, experiences with family, professionals, and teachers while parenting a child with autism; and the main focus was on the third group related to respondents’ relationship with religion and spirituality vis-à-vis autism. They were asked about their religious background and beliefs as well as about experiences with religion in the context of autism for their children. Additionally, two religious educators running a specialized program for children with autism SPRED were interviewed on-site	Forty-seven parents and six young adult siblings of children and youth with mod-severe autism	Overall results indicated that parents who valued religion themselves attempted to share and transmit it to their children and believed that it is beneficial to their children and youth with autism. A total of 24 out of 53 had tried formal religious education at a congregation for their child or youth with autism, whereas three children had attended a religious school. The remaining 26 had various reasons for not attempting religious education for them. Some were not religiously inclined themselves, whereas others believed that it would not benefit them, would be too difficult, or would be beyond their ability to understand, or attend religious education. Of the 24 participants who received congregational education, 11 reported positive experiences, eight reported negative experiences, and eight were noncommittal. About half of the respondents had not pursued religious education for their child because they thought that either their child did not need it or would not benefit from it. Details of a visit to a SPRED in the United States where five children were being educated were shared. The program had modified various environmental factors to fit the attendee's needs and was hugely appreciated by the parents	A varied sample from New England and the southern part of the United States was represented. New insights into how children and youth with autism experience religion in religious education from their parents’ perspective in depth. Verbatim, our specific target population’s parents gave valuable insights about the topic under study. Expressions of parents of two 16 years with autism emphasized the role of religious leaders and teachers in the experiences and attendance of autistic adolescents where one described them as being unknowledgeable about her disabilities and another appreciating his “openness and acceptance”	A mixed sample from the age range of 4-29 years with a median age of 12. More number of girls (46). The majority of respondents (26/40) who reported a religious affiliation were Catholic, while 14 were affiliated with other churches. Two were Jewish. 77% were White. The IQ level of these children was not available. It was the parents’ perspective not firsthand

Religion is a multidimensional concept that expands over several life domains: social, personal, and emotional. It is understandable, therefore, that a certain set of basic skills would be required to be able to practice religion in all its wholesomeness. Autism is a condition characterized by a lack of social communication and reciprocation skills. Research suggests that autistic individuals have difficulty understanding others’ mental states and empathizing with them [36]. Most religious systems usually involve concepts such as belief in supernatural agents and agency, some degree of understanding of others' mental states, as well as imagination and empathy, which would be required to practice those concepts. The various religious developmental theories also emphasize the importance of these concepts, such as anthropomorphism [54], theory of mind (ToM), and empathy [55], in developing religiosity. So, it would not be improbable to logically assume that autistic individuals might have subdued religiosity as compared to NT individuals owing to their decreased cognitive abilities. The following studies explored religiosity, spirituality, or related concepts in the context of cognitive abilities like mentalizing, empathizing, or imagination [14,29,31,32,44,45].

The first breakthrough study focusing on religiosity and autism in adolescents in the context of mentalization was published in 2012. Out of a group of studies, study 1 recruited young autistic individuals and found that they had a lower score on belief as compared to NT adolescents [29]. This was a landmark study that inspired further research. The study was well-designed, using adaptations of validated tools for religiosity and mentalization, and included participants from various religious beliefs. The comparison group was well-matched for various demographic and social characteristics. However, the sample size was small, consisting of 11 autistic and 13 NT adolescents, with only one female in each group. Religiosity was assessed using a self-reported four-item measure, but the proposed predictors of religiosity, that is, autism quotient (AQ) and empathy, were parent-reported. Another noticeable limitation was that despite a small sample size, individual ages or age ranges of the sample were not provided, and the mean ages recorded were slightly different for both groups. Overall, the study concluded that IQ was not a predictor of religiosity, but mentalization (AQ and empathy) was. Since it has been proposed that gender might be related to religiosity [56], the probability of results being skewed from this small sample size with only one female cannot be excluded.

In 2016, researchers carried out work on young people with HFA [14], where they matched young autistic individuals with NT controls (N = 19 in each group; male-to-female ratio 15:4) in the age range of 12-31 years; however, associations were calculated after controlling for age, IQ, and religious upbringing. They assessed seven major domains of religiosity (adaptation of validated instruments, internally validated for research) and used various mentalizing methods like strange stories and false belief tasks to find the association between the two. Overall, there was no statistically significant difference between the two groups. Autistic adolescents, however, did have a statistically significant lower attraction to prayer. He concluded that HFAs have sufficient cognitive abilities to think and communicate with God and that any cognitive impairment they may have has little effect on other aspects of their religiosity besides their attraction to prayer.

A similar study [29] was replicated five years later by other fellow researchers [45] who attempted to improve on the previous methodology. Study 5 out of a group of studies included 33 HFA adolescents and 29 NT controls, out of whom 28 and 17 were males, respectively. Not only were the data collected from the participants themselves, but a mix of various techniques, including self-reports and intentional videos such as geometric figure task (GFT), were used to measure the association between religiosity and mentalization. Additionally, the role of cultural factors in religiosity was assessed. The study concluded that the groups did not differ on religious behaviors, religiosity, or credibility-enhancing displays (CREDs) and that mentalizing difficulties could not be linked with disbelief. One significant limitation, though, was that the religiosity measure was not validated. The research highlighted the postulation that factors like CREDs of religiosity might play a bigger role in determining one’s views and beliefs than innate cognitive features.

An interesting finding in a small sample size comparison study in a predominantly Catholic school found that the autistic adolescents scored almost the same as NT controls on factors like religious practices and perceptions of God, but their ability to attribute agency to supernatural beings decreased as they grew older [32]. A mixed-methods study by Visuri, while studying the role of empathy as a part of mentalizing ability in the development of religious beliefs, found no statistically significant difference in empathy concern scores between autistic adolescents and NT controls [31]. Yet another study concluded that autistic adolescents achieved significantly higher mean religious experience scores than their NT controls [48].

In three qualitative studies, HFA adolescents were interviewed, and they narrated their experiences regarding religion, faith, and their importance in their lives in their own words [44]. In the second study [31], young autistic people expanded on various supernatural beliefs and faiths about bodiless agents. All participants affirmed having invisible companions. Most talked about being able to shift back and forth between the real world and imagination in the form of games, books, and daydreaming. It can be concluded that participants were imaginative and found the bodiless agents predictable and emotionally coherent contrary to the widespread belief that autistic individuals might perceive mental representations of superhuman agents as puzzling [31]. In one interview, based on a Swedish background, most of the autistic adolescents confirmed experiencing supernatural experiences in somatosensory modalities [48]. They seemed to use supernatural attributions as a coping strategy. Many ascribed this to invisible agents outside organized religion. This was an interesting finding that is in line with previous religious developmental theories, which postulate that religion is a social field and culture and society play a significant role in shaping it [57]. This might explain how culture, occulture, and popular media can influence supernatural beliefs. The other available supernatural alternatives in popular media or culture may fill the gap left when there is a decline in religious stories with supernatural contexts in relatively secularized societies.

Findings from these studies clearly show that the cognitive ability of autistic adolescents, if not higher (as found in a few domains), is at least comparable to, or at the very least not too low, to affect their ability to understand or practice religious or supernatural concepts. However, all these studies have been done with a subset of autistic adolescents (HFA). Therefore, it might be justifiable to say that, in light of this review, religiosity (in terms of cognitive ability) might not be significantly subdued in a subset of autistic adolescents (HFA). However, the same statement might not be true for the autistic adolescent population as a whole, where only about half of them may have an average or above-average IQ [58].

These results are contrary to the findings in previous research that found a negative association between religiosity and autism [59]; however, the correlation was weak, and it was further explained that autistic symptoms would not necessarily point to atheism. Another study suggested that HFA individuals were more likely to identify as atheists and that AQ in atheists was higher than that in religious groups [36]. That study collected data from internet religious forms as an online survey. The participants were not physician-diagnosed, and the authenticity of the data was questionable. The results were in line with the findings from research that concluded that mentalization was either negatively related to or unrelated to religiosity in an NT college student sample [60].

The results do not defy the classical religious developmental theories that emphasize the importance of theory of mind (ToM), empathy [55], and anthropomorphism [54]; however, they propose that in HFA individuals, these abilities are not so compromised as to affect their ability to be religious; and minor deficiencies in these cognitive abilities would not significantly affect the ability of HFA adolescents to practice religiosity.

Most studies found no significant difference between autistic adolescents and NT adolescents in observable behaviors like service or congregational attendance, which require physical effort and social skills to some degree. A parent-report survey of children and adolescents with intellectual disabilities (IDs) and autism found no association between autism and spiritual practices [49]. Two comparison studies found no difference in religious behavior [45] or religious practices [32] between autistic adolescents and control groups.

Out of two qualitative studies with participants from religious backgrounds, all participants in one study reported attending worship services weekly except one, who did so monthly [44], while the other reported that nearly half of the autistic adolescents attended some form of religious education/congregation [28].

One study, though finding no difference in the frequency of prayers between the two groups, did find a statistically significant difference in the "attraction to prayer" between the two groups [14]. Greater attraction to prayer was associated, however, with lower deficits in social skills and could be linked to difficulties in social interaction in general. However, most of this research (4/6) was not controlled for religious affiliation or background. Two parent-reported surveys found fewer congregational activities in a mixed sample of autistic adolescents and ID [49] and a 50% decrease in religious service attendance in autistic adolescents [47].

Although most of the research shows no significant difference between autistic adolescents and NT adolescents regarding religious practices, the results need to be interpreted with caution, considering that most of the participants came from religious backgrounds. In the review itself, it was found that parental religious beliefs and activities were associated with the autistic youth's religious beliefs and practices [49], and parents who valued religion themselves attempted to share or transmit it to their children [28]. These findings align with previous research, suggesting that parents, peers, and schools influenced religious service attendance in NT adolescents [61].

The study by Reddish et al. [14] was controlled for religious affiliation and did show a statistically significant difference in "attraction to prayer." The study by Lee et al. had the largest sample size (173 autism vs 23,768 controls) and the most widespread recruitment (50 US states) targeting adolescents specifically, so the results of a 50% decrease in religious service attendance are hard to ignore. Subjects were controlled for the absence of disabilities. The study did not target HFAs only, unlike most of the other studies [47]. Therefore, stating that religiosity (in terms of religious behavior) was almost the same (HFAs) to somewhat mildly reserved (overall autism group) in autistic adolescents as compared to NT controls would be justifiable in light of this review.

The religious experience appeared as another prominent theme related to religiosity in autistic adolescents in this review. In a qualitative review [44], the adolescents reported that they viewed faith as a positive influence in their lives. In terms of sensory and supernatural religious and spiritual experiences, they scored higher on mean religious experience scores, as well as "felt presence of God" as compared to the control group [48]. All described interactions with bodiless agents, most citing God as their invisible companion [31]. They expressed their imaginative sides and said it was easier for them to interact with supernatural beings than humans. They expressed empathy. They admitted to having various somatosensory and spiritual experiences and being interested in supernatural beings outside of traditional organized religions. They used immersion in the imagination through stories, video games, and daydreaming as coping and problem-solving mechanisms [31,48].

In light of the findings, it can be concluded that autistic adolescents do not have a significantly reserved religiosity (religious and supernatural experience), but again, these findings represent one subset of autism only (HFA) and cannot be generalized to the whole group. So far, the current research is insufficient to answer this question for the whole group, as research in this domain is only carried out on HFA and those with verbal skills, due to the difficulties in expression experienced by nonverbal or below-average IQ individuals with autism.

This result was understandable from the point of view of religious developmental theories as well, which postulate that cognitive abilities about supernatural agents and agency are an important aspect of religious cognition [62]. The HFA participants under discussion experienced a wide range of supernatural experiences and were found to have the same degree of religious experience as NT adolescents. Thus, looking at the concept of religiosity and spirituality as a whole, it can be inferred from the available research included in this review that religiosity (cognitive abilities, behaviors, and experiences) in a subset of adolescents with autism (HFA) might not be significantly reserved as compared to NT adolescents. However, there is not enough research to conclude the same, or indeed the opposite, for autistic adolescents in general.

The little research available on autistic individuals as a whole (and not exclusively on a subset of HFA individuals) is mainly based on observable behaviors, such as church, mass, or congregational attendance, possibly due to the methodological limitations of assessing other domains in individuals with intellectual or verbal deficits. The level of religiosity (based on religious attendance) was found to be lower in this population than the NT controls in one study [47].

Parent and caregiver interviews highlighted somewhat similar themes overall in qualitative interviews. The majority considered religious inclusion and participation beneficial and essential for their children and wanted them to know and learn their beliefs. They expected stakeholders like religious bodies, the government, and teachers to include and support them in achieving this. Only a few had negative experiences with the religious services, and some thought that either their children did not need them or they would not benefit from them. Similar ideas are being supported in upcoming research proposing that families with disabled children may find a path to full social inclusion through religious organizations [63]. This aligns with Elkind's study of religious development, which postulates that institutions such as religion help individuals in society as they move through different phases of life to find solutions for various problems they encounter [64].

The review highlighted the possible relationship between quality of life (QoL), faith, and religiosity. Autism was associated with low faith and poor QoL. These findings are in line with previous findings suggesting that autistic individuals have a much lower QoL than NT individuals [65].

Identifying unique and relevant resources for this vulnerable and much-needed patient population is essential. Providing support in this area for families and their children could prove helpful and improve the QoL of these adolescents with disabilities. Research on autistic adults has shown that they not only have spiritual needs but also use religious and spiritual practices and that these elements are connected to several life-quality indicators [66].

Significance and implications

Autistic individuals and their caregivers regard religiosity and spirituality as important domains in both their and their children's lives. They want their children to be given opportunities to be a part of religious groups and look forward to the government, religious, and healthcare authorities actively supporting them in this area. The QoL of these adolescents is positively associated with their strength of faith. This can have implications for strategies to improve their QoL. Adolescence is a transitional time. It is a critical period of discovering oneself and deciding on actions that will probably affect one for the majority of one's adult life. The decision to pursue a certain career, adopt a certain set of beliefs, or generally define one's values often comes at this time. This vital period of life, with its stresses combined with the burden of neurodiverse conditions like autism, multiplies the struggle. Any support, like religion/spirituality, that might serve as an additional resource for the adolescent and the family would be appreciated.

Looking at the multidimensional concept of religiosity, which encompasses the social, cultural, and personal domains, and its capability of engaging multiple disciplines in an individual's life, it seems to have a promising potential to be used as a resource for many, if not all, autistic adolescents. The findings call for policymakers, religious leaders, and healthcare professionals to devise strategies for inclusion and support for autistic adolescents. The possible role of religion as a resource and coping strategy for these children and their families is worth exploring.

Limitations

This review had a few limitations. All the included studies were cross-sectional. Some studies lacked methodological rigor and had biases such as small sample sizes and a lack of controls for possible confounding factors, such as IQ, gender, and religious affiliations. Most studies were not largely generalizable and mainly represented the Western world. Some studies were performed on a mixed population including adolescents and adults or children. Caution was taken to include the results from such pieces of research where either calculation was done after age was controlled or separate analyses were done for separate age ranges. Where this was not possible, the result was discussed, with the respective limitations mentioned. Most of the studies did not use adolescent-specific measures or tools for measuring religiosity or spirituality, accounting for relevant issues like age-appropriate language or parental religiosity.

Recommendations for further research

Since the study by Visuri [48] in 2020, no further significant research focusing on religiosity in autistic adolescents has been conducted. Research focusing on the comparison of a sample of autistic adolescents, with and without ID, with or without physical disability, and with NT controls can provide significant insight and bridge existing knowledge gaps. The use of validated, developmentally adapted measures to assess religiosity domains as well as advanced mentalizing measures and techniques like geometric figure videos and CRED scales while controlling for factors like age, gender, and religious affiliation might notably increase the validity of future research in this area. A better study design, like a longitudinal follow-up approach from childhood to adulthood, could be more insightful in understanding this developing and evolving phenomenon of religiosity in this population group. Further research on the impact of religiosity and spirituality on QoL, adjustment, and coping with disability for autistic adolescents and their families, using religion and spirituality as a resource, can prove to be a promising area.

## Conclusions

The review provided a deeper understanding of religiosity, its development, and expression in autistic adolescents compared to NT adolescents in a multidimensional context. The emerging themes highlighted the fact that religiosity, faith, spirituality, and related concepts manifest in various dimensions and sociocultural factors and are not dependent on the individual’s state alone.

Summary of the conclusion

Below is a summary of the most significant findings.

The ability to practice religion, owing to cognitive abilities such as mentalization and empathy, might be limited but not absent in the autistic adolescents under review, contrary to common beliefs. Being religious or spiritual might require a basic cognitive ability and some degree of theory of mind; however, this is neither fundamental nor the sole predictor of this multidimensional concept. Autistic adolescents had comparable empathy to their NT controls. HFA individuals were highly imaginative, and modern media and occulture played a significant role in shaping their supernatural beliefs and agency. HFAs found it more comfortable to communicate with supernatural agents, perhaps due to their coherent, controlled, and less complicated cognitive styles, perhaps without having to abide by NT social standards and norms.

Observable religiosity, such as religious attendance, was low compared to the NT adolescent sample in one large national survey. However, most of the studies suggested no significant difference between the two groups. Where religious attendance was found to be low in a large autistic adolescent sample compared to controls, the carer burden was high. This calls for investigating the role of carer burden as a possible limiting factor for religious service attendance. Greater attraction to prayer was associated with lower deficits in social skills and could be linked to struggles in social interaction in general. Culture and society played a prominent role in shaping religiosity and spirituality in these adolescents through CREDs of religiousness or atheism/secularism. Factors like religious background, affiliation, and the parents' or caregivers' strength of faith had a significant impact on the autistic adolescent’s religiosity.

Most caregivers wanted their children to learn about religion and encourage spirituality, believing that it was important in their children's lives. The opinions of carers assume utmost significance in this context because, for teenagers, families will play the most active role in promoting religious and spiritual expressions until they move to adulthood. Reserved religiosity could be attributed to many factors, and decreased mental ability/mentalization, empathy, or imagination did not seem to be the primary predictors or contributors to the development of religiosity in this population. The role of culture, parents, caregivers, and religious affiliations was significant. It might prove to be a stronger contributor to religiosity and its expression than other previously argued predictors like mentalization. QoL was positively associated with faith in autistic adolescents.
